# Nosocomial Outbreak of Parechovirus 3 Infection among Newborns, Austria, 2014

**DOI:** 10.3201/eid2209.151497

**Published:** 2016-09

**Authors:** Volker Strenger, Sabine Diedrich, Sindy Boettcher, Susanne Richter, Peter Maritschnegg, Dietmar Gangl, Simone Fuchs, Gernot Grangl, Bernhard Resch, Berndt Urlesberger

**Affiliations:** Medical University of Graz, Graz, Austria (V. Strenger, P. Maritschnegg, S. Fuchs, G. Grangl, B. Resch, B. Urlesberger);; Robert Koch-Institute, Berlin, Germany (S. Diedrich, S. Boettcher);; Institute for Veterinary Disease Control, Mödling, Austria (S. Richter);; Regional Hospital Feldbach, Feldbach, Austria (D. Gangl)

**Keywords:** Human parechovirus type 3, sepsis-like illness, nosocomial infections, parechoviruses, picornaviruses, viruses, Austria

## Abstract

In 2014, sepsis-like illness affected 9 full-term newborns in 1 hospital in Austria. Although results of initial microbiological testing were negative, electron microscopy identified picornavirus. Archived serum samples and feces obtained after discharge were positive by PCR for human parechovirus 3. This infection should be included in differential diagnoses of sepsis-like illness in newborns.

Parechoviruses are small, nonenveloped, single-stranded RNA viruses belonging to the family *Picornaviridae*. Although most human parechovirus (HPeV) infections cause self-limiting mild respiratory or gastrointestinal symptoms, HPeV type 3 (HPeV3) has been found in 5%–13% of newborns and young infants <3 months of age with late-onset sepsis or encephalitis (*1*–*12*). Knowledge of HPeV3 infections originates from single cases or small series of sporadic unrelated infections. One considerable outbreak, affecting ≈200 infants with obviously community-acquired diseases, was recently reported from Australia (*1*,*2*). In contrast, we describe a timely confined, and apparently nosocomial, outbreak of HPeV3 infection originating from 1 maternity ward in Austria, affecting one fifth of newborns hospitalized during that period. 

## The Study

During August 5–September 7, 2014, a total of 9 newborns, 2–27 (median 5) days of age, showed signs of sepsis-like illness (fever and reduced general condition) and were admitted to the Department of Paediatrics and Adolescent Medicine, Medical University of Graz, Graz, Austria. All had been delivered within 2 weeks in the same obstetric unit at the Regional Hospital Feldbach, which is located in a small town (4,500 inhabitants) in southeastern Styria, a region of southern Austria of which Graz is the capital. During these 2 weeks, 44 newborns had been delivered at this obstetric unit (total 1,400/y); 9 (20.5%) born during these 2 weeks became symptomatic (Table; Figure). Diagnostic procedures were performed at the discretion of each attending physician and comprised testing for adenovirus, enterovirus, norovirus, rotavirus, herpesvirus 1, herpesvirus 2, varicella zoster virus, Epstein-Barr virus, cytomegalovirus, human herpesvirus 6, and parvovirus B19. However, no causative agent was identified.

**Table:** Clinical characteristics for 9 newborns with human parechovirus 3 infection, Austria, 2014*

Characteristic	No. (%) patients	Median (range)†
Demographic		
Male sex	5 (55.6)	NA
Gestational age, wk + d	NA	40 + 1 (38 + 3 to 41 + 2)
Birth weight, g	NA	3,480 (3,240–4,160)
Vaginal delivery	6 (66.7)	
Age at onset of fever, d	NA	6 (1–27)
Age at admission to PD, d	NA	6 (2–27)
Time from discharge from maternity to admission to PD, d	NA	1 (0–24)
Clinical		
Fever	9 (100)	39.1°C (38.5°C–39.9°C)
Reduced general condition	4 (44.4)	NA
Tachypnea	4 (44.4)	NA
Tachycardia	3 (33.3)	NA
Drinking difficulties	5 (55.6)	NA
Circulatory centralization	3 (33.3)	NA
Increased irritability	3 (33.3)	NA
Treatment		
Admission to NICU or PICU	3 (33.3)	NA
Antimicrobial drug treatment (cefuroxime + amoxicillin)	8 (88.9)	3 (2−5) d
Intravenous hydration	4 (44.4)	NA
Length of stay at PD, d	NA	3 (0−9)
Laboratory values		
Leukocytes, cells/μL	NA	7,690 (4,750–18,310)
Granulocytes, cells/μL	NA	4,595 (1,300–10,124)
Lymphocytes, cells/μL	NA	2,330 (590–7,100)
Lymphopenia, <2,000 cells/μL	4 (44.4)	NA
Monocytes, cells/μL	NA	1,100 (660–2,470)
Minimal thrombocytes/μL	NA	200,000 (101,000–270,000)
C-reactive protein, mg/L‡	NA	1.6 (<0.6–11.4)
C-reactive protein >5 mg/L	2 (22.2)	NA
Procallcitonin, ng/mL§	NA	0.18, 0.18, and 0.36
Interleukin-6, pg/mL¶	NA	21.0 and 124.8

*NA, not applicable; NICU, neonatal intensive care unit; PD, pediatric department; PICU, pediatric intensive care unit. †Unless otherwise indicated. ‡Upper limit of reference range = 5.0 mg/L. §Upper limit of reference range = 0.5 ng/mL; measured for 3 patients. ¶Upper limit of reference range = 7.0 pg/m; measured for 2 patients.

**Figure:**
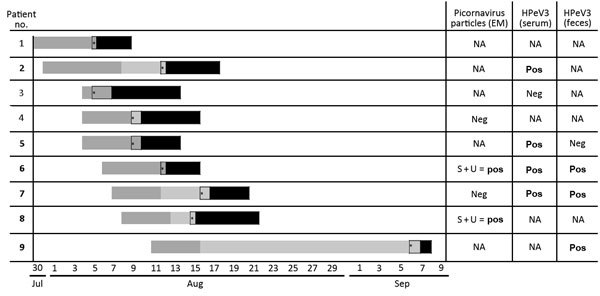
Chronological details for 9 newborns with human parechovirus 3 infection, Austria, 2014. Either human parechovirus 3 (HPeV3; detected by real-time reverse transcription PCR, n = 5) or particles resembling picornavirus (detected by electron microscopy [EM], n = 2) were detected in >1 of the analyzed materials for 6 (indicated in boldface) of 8 patients. For 1 patient, neither EM nor PCR had been performed. Dark gray bar, postdelivery stay in maternity ward; light gray bar, stay at home; black bar, stay at pediatric department. *Onset of fever. NA, not applicable; neg, negative; pos, positive; S, serum; U, urine.

Because the only pediatric department in southern Styria (including Feldbach) is at the Medical University of Graz, all severely ill newborns or infants are admitted there. However, no additional cases of sepsis or sepsis-like illness of unclear etiology were identified in newborns or young infants from that area during these months. The facts that the outbreak was temporally confined and all affected patients had been nursed in the same ward strongly indicated a common source within this unit. Investigations to detect a possible common source in the affected obstetric unit comprised anamnestic and clinical examination of hospital staff members and the newborns’ mothers, surface swabbing (e.g. nursery rooms, baby baths, examination beds, diaper changing tables), microbiological examinations of formula, and analysis of staff roster and occupancy plans. However, a presumed common source or causative agent could not be identified.

To further seek the causative pathogen, we analyzed serum, urine, and nasal secretions from 4 patients (nos. 4, 6, 7, 8; Figure) by negative staining in a transmission electron microscope (Zeiss 906, Oberkochen, Germany) at 80 kv. Although nasal secretions revealed no particles, serum and urine of 2 of the tested patients (nos. 6, 8) contained icosahedral particles, diameter 20–30 nm, resembling picornaviruses. Because of the young ages of the patients, we assumed that the particles were HPeVs. However, by the time we received the electron microscope results indicating picornaviruses, all affected patients had been discharged. We therefore requested fecal samples from all 9 discharged patients for molecular diagnostics of HPeV. We received samples from 4 patients (nos. 5, 6, 7, 9) a median of 29 days (range 9–34) after discharge. 

RNA was isolated by using a QIAamp Viral RNA Mini Kit (QIAGEN, Hilden, Germany) according to the manufacturer’s instructions. For HPeV detection, we used a real-time reverse transcription PCR (RT-PCR) selective for the 5′ nontranslated region as described (*13*,*14*). Molecular typing of positive samples was conducted by nested real-time RT-PCR and sequencing of the partial capsid viral protein (VP) 3/VP1 region by using primers described by Harvala et al. (*15*). We performed reverse transcription and first-round amplification by using a One-Step RT-PCR Kit (QIAGEN). Nested amplification was performed by using a HotStarTaq Master Mix Kit (QIAGEN). The resulting fragment (≈300 bp) was subsequently sequenced by using a BigDye Terminator v.3.1 Cycle Sequencing Kit (Applied Biosystems/Life Technologies, Darmstadt, Germany) and nested PCR primers. Sequences were aligned to reference sequences in GenBank by using a BLAST (http://blast.ncbi.nlm.nih.gov//Blast.cgi) algorithm (Technical Appendix). 

Of the 4 fecal samples tested, 3 were positive for HPeV. Molecular typing that used partial VP3/VP1 capsid protein regions revealed HPeV3 in all 3 samples. To analyze the presence of this pathogen even during acute illness, we retrieved archived serum samples from 5 patients (nos. 2, 3, 5, 6, 7; Figure). No samples had been retained for 3 other patients. For 4 of the 5 patients tested, archived serum samples were positive for HPeV-3, indicating systemic infection with HPeV3 during the symptomatic phase of disease. Comparison of all 7 sequences (from 3 fecal samples and 4 serum samples) revealed 100% identity. Resulting sequences were submitted to GenBank (accession nos. KU556748–KU556754).

Clinical signs and symptoms and laboratory changes for these 9 patients were compatible with those published for patients with HPeV3 infection (*1*,*2*). All 9 newborns recovered without complications; no severe, long-term complications were noted 15 months later.

Despite intensive epidemiologic evaluation, we were not able to identify a human (personnel, mothers, siblings, or visitors) or nonhuman (surfaces, formula) source of infection within this ward. However, because the causative agent was identified several weeks after the end of the outbreak, testing for HPeV had not been performed on any such human or environmental specimens. Therefore, we are not able to clearly differentiate common-source infection from person-to-person transmission. Because newborns do not have direct contact with each other, an asymptomatic adult or older sibling might have been the unidentified common source. This hypothesis is in line with the fact that most infections with HPeV in adults are asymptomatic. Of the 9 newborns, 4 became symptomatic while still hospitalized; thus, they were certainly infected while in the maternity ward. The other 5 became symptomatic after discharge from the obstetric unit, so they might have acquired the infection while outside the hospital. However, the interval between discharge and readmission was <1 week for all but 1 patient, and no cases of community-acquired sepsis and sepsis-like illness in newborns or infants from that region without association with the affected maternity ward could be identified during that period. 

Thus, the infection was most likely nosocomial for at least 8 of the 9 patients. Assuming the period of infection for these patients (i.e., during their stay in the maternity ward), we can draw conclusions with regard to the incubation period of HPeV3 infections. The observed intervals between infection and appearance of symptoms were from 1 to 12 days. Only patient 9 became symptomatic 27 days after discharge; this patient might have acquired the infection outside the hospital.

## Conclusions

For newborns, HPeV3 is a relevant pathogen; febrile illness appears as sepsis. After symptomatic infection, a newborn can shed HPeV3 in feces for at least 1 month. The contagious nature of the virus can lead to nosocomial outbreaks. Thus, timely identification of the causative agent may prevent nosocomial transmission (by isolation and identification of the source) and unnecessary treatment with antimicrobial drugs. For newborns with sepsis-like illness, routine diagnostic considerations should include HPeV3.

Technical Appendix Real-time reverse transcription PCR and amplification of the partial capsid protein (VP) 3/VP1 junction region for molecular typing of parechoviruses, sequencing, and primers used.
