# Supplementary material for: Nosocomial Outbreak of Parechovirus 3 Infection among Newborns, Austria, 2014
Source: Emerg Infect Dis. 2016 Sep;22(9):1631–4. doi: 10.3201/eid2209.151497 (PMC4994342; doi:10.3201/eid2209.151497)
Supplement: Technical Appendix — Real-time reverse transcription PCR and amplification of the partial capsid protein (VP) 3/VP1 junction region for molecular typing of parechoviruses, sequencing, and primers used. [file 15-1497-Techapp-s1.pdf]

Article DOI: http://dx.doi.org/10.3201/eid2209.151497

Nosocomial Outbreak of Parechovirus 3 Infection among Newborns, Austria, 2014

Technical Appendix

Real-time RT-PCR and Amplification of VP3/VP1 Junction Region for Molecular Typing of Parechoviruses

Screening for parechovirus was done by realtime RT-PCR with primers described in Benshop et al. 2008. Briefly, the Superscript III Platinum One-step Quantitative RT-PCR system by Invitrogen (Darmstadt, Germany) was used as described in the manufactures protocol. For each sample, a doubled reaction mix was prepared containing 300 nM of primers Parecho F31 (NRZ 202) and K30 (NRZ 203) as well as 150 nM of probe HPeV-WT (NRZ-TM2). Reverese transcription was carried out for 15 min at 50°C followed by 2 min 95°C for denaturation. Amplification was done in 50 cycles 15 sec 95 and 35 sec 60°C.

Partial VP1 was amplified by using the One-Step-Reverse Transcription PCR Kit (QIAGEN), followed by a nested PCR with HotStarTaq-Mastermix (QIAGEN). Reverse transcription PCR was conducted in a 12.5-µL reaction that contained 2 µL of RNA, 500 nM of primer NRZ 193 500 nM of NRZ 194 according to the manufacturer's protocol. The temperature profile used was 43°C for 60 min, (53°C for 60 sec, 55°C 60 sec)x20, 70°C for 15 min and 95°C for 15 min, followed by 35 cycles at 94°C for 30 s, 50°C for 30 s, and 72°C for 90 s. Final elongation conducted at 72°C for 10 min.

Two semi-nested PCRs were conducted by using 1 µL of reverse transcription PCR samples in a 12.5-µL volume that contained 500 nM of primer NRZ 193 and 500 nM of primer NRZ 196 as well as 500 nM of primer NRZ 195 and 500 nM of primer NRZ 194. Amplification was conducted by 35 cycles (94°C for 30 s, 55°C for 40 s, and 72°C for 40 s), and final elongation 72°C for 10 min. The resulting amplification products (368 bp and 364 bp) were visualized by electrophoresis on a 1.5% agarose gel.

Sequencing

Partial VP3/VP1 amplification products were used directly for sequencing. Sequence reaction was conducted by using primers NRZ 194, NRZ 195, and NRZ 196. Resulting

sequences were submitted to GenBank under following accession numbers: KU556748 - KU556754

Technical Appendix Table. Primers used for reverse transcription PCR to detect human parechovirus

Primer/probe	Sequence, 5′→3′	Position in reference strain AB084913	Reference	Reference name
NRZ 202	CTGGGGCCAAAAGCCA	429–444	(*1*)	ParechoF31
NRZ 203	GGTACCTTCTGGGCATCCTTC	569–549	(*1*)	K30
NRZ TM2	**VIC**-MGB-AAACACTAGTTGTA(A/T)GGCCC-**BBQ**	527–546	(*1*)	HPeV-WT
NRZ 193	GAYAATGCYATMTAYACWATYTGTGA	2089–2114	(*2*)	2019
NRZ 194	ACWGTRAARATRTCHACATTSATDG	2522–2498	(*2*)	2523
NRZ 195	TTYTCMACHTGGATGMGGAARAC	2158–2180	(*2*)	2159
NRZ 196	DGGYCCATCATCYTGWGCTGA	2457–2430	(*2*)	2458

References

1. Benschop KS, de Vries M, Minnaar RP, Stanway G, van der Hoek L, Wolthers KC, et al. Comprehensive full-length sequence analyses of human parechoviruses: diversity and recombination. J Gen Virol. 2010;91:145–54. PubMed http://dx.doi.org/10.1099/vir.0.014670-0

2. Harvala H, Robertson I, McWilliam Leitch EC, Benschop K, Wolthers KC, Templeton K, et al. Epidemiology and clinical associations of human parechovirus respiratory infections. J Clin Microbiol. 2008;46:3446–53. PubMed http://dx.doi.org/10.1128/JCM.01207-08
